# Ambulances are for emergencies: shifting attitudes through a research-informed behaviour change campaign

**DOI:** 10.1186/s12961-019-0430-5

**Published:** 2019-03-28

**Authors:** Kim Borg, Breanna Wright, Liz Sannen, David Dumas, Tony Walker, Peter Bragge

**Affiliations:** 1 BehaviourWorks Australia, Monash Sustainable Development Institute, Monash University, 8 Scenic Boulevard, Clayton, Victoria 3800 Australia; 2 Victorian Department of Health and Human Services, Melbourne, Australia; 3 The Shannon Company, Melbourne, Australia; 4 Ambulance Victoria, Melbourne, Australia

**Keywords:** Ambulance, mass media, behaviour change, attitudes, emergency service

## Abstract

**Background:**

In Victoria, Australia, emergency calls requesting an ambulance have been increasing at a rate higher than population growth. While most of these calls are for genuine emergencies, many do not require an immediate ambulance response. A collaborative research approach was undertaken to address this issue. The aim of this paper was to evaluate the effectiveness of applying a behaviour change approach to this challenge by first addressing antecedents of behaviour (attitudes, awareness and knowledge).

**Methods:**

The project included a formative research phase to inform the design of a mass media campaign and subsequent evaluation of the campaign.

**Results:**

Results indicated that the campaign was successful in increasing community attitudes towards ambulances as being for emergencies only, particularly among those familiar with the campaign material and with other health service options (such as telephone advice lines).

**Conclusions:**

These findings provide support for adopting the Forum approach to increase the chances that a mass media campaign will achieve its stated objectives. Recommendations for future campaign activities are discussed.

**Electronic supplementary material:**

The online version of this article (10.1186/s12961-019-0430-5) contains supplementary material, which is available to authorized users.

## Background

Ambulance Victoria (AV) provides emergency ambulance coverage across the state of Victoria – Australia’s second-most populous state [1]. AV responds to over 840,000 emergency and non-emergency cases, transporting over 660,000 patients via road or air each year [2]. Triple Zero (000) is the emergency services number throughout Australia for ambulance, fire and police, with 40% of calls to Triple Zero being from individuals requesting an ambulance [3].

In recent years, demand for ambulances in Victoria has been increasing faster than the rate of population growth, and many of those calls are not associated with a genuine emergency [4, 5]. A large number of Triple Zero calls are managed without dispatching an emergency ambulance, for example, by utilising non-emergency transport options, referring the caller to alternative health services, recommending self-presentation to a hospital Emergency Department, or providing self-care advice [3]. However, the use of limited Triple Zero and AV resources to deal with non-emergency calls reduces availability of ambulances to quickly respond to patients experiencing life-threatening emergencies [6]. Reducing the number of calls or attendance cases that do not require emergency attention would therefore relieve pressure on emergency medical services. Therefore, a major review of Victoria’s ambulance services (released in December 2015) identified the need to improve public awareness of the role of ambulance services and when it is appropriate to dial Triple Zero [5].

### Changes to ambulance services in Victoria

In 2015, changes were made to the Victorian ambulance system including how AV is funded, how paramedics are supported through their careers, and how Triple Zero calls are triaged and ambulances dispatched. A revised Clinical Response Model was implemented, which focused on the individual needs of patients and the most appropriate response for those patients to ensure ambulances are available for emergencies. Under the revised Clinical Response Model, some events are now being assessed more thoroughly through a secondary triage process by expert paramedics and registered nurses in the AV Referral Service. This service ensures that genuine emergencies are identified and get an emergency response, and that non-emergencies can be safely and appropriately referred to alternative transport options and health services to better meet their needs. As a result of this strategy, an estimated 7000 additional emergency patients now receive paramedic care within the 15-min response target each year [7].

In 2016, the Victorian Government’s Department of Health and Human Services allocated funding for additional paramedics and associated infrastructure to further improve ambulance response times [7]. To further address demand, they also announced a new initiative to establish 20 community pharmacies open over 24 h by mid-2018. These ‘Supercare pharmacies’ are staffed by registered nurses in the evening and provide additional healthcare options.^1^ The Department of Health and Human Services also commissioned a communications campaign to ultimately reduce inappropriate calls to Triple Zero for ambulances and increase the use of other appropriate health services.

The aim of this research was to evaluate the effectiveness of applying a behaviour change approach to this challenge by first shifting attitudes and increasing awareness and knowledge regarding ambulance use in the state of Victoria. Specifically, this research sought to evaluate the extent to which the Victorian community viewed ambulances as an emergency service before and after the campaign.

### The current study

This study comprised two research phases, namely a formative phase and a campaign development and evaluation phase. Ethics approval was obtained from Monash University’s Human Research Ethics Committee for both research phases. The methods and results of these two phases are presented separately as the second phase drew upon findings from the first.

## Phase 1 – Formative research

### Methods

For the formative research, the Forum approach of combining a rapid evidence and practice review with a structured stakeholder dialogue was used [8]. Established in Canada in 2009 and pioneered in Australia in 2012, the Forum approach has demonstrated high participant satisfaction and intentions to act [8]. It centres around two key activities, involving a rapid evidence and practice review and a structured stakeholder dialogue.

#### Rapid evidence and practice review

Unlike traditional systematic literature reviews, rapid reviews focus on synthesised research evidence and/or high-quality or recent primary studies [9]. The addition of semi-structured interviews adds a critical dimension to the academic literature review. Where literature answers the question ‘what does the evidence say?’, interviews can address the question ‘what’s happening in practice and how will this affect implementation?’; both perspectives are useful in a policy-making context, especially if the intersection between evidence and practice is able to be identified, as demonstrated by previous published Forum projects [10–12].

This rapid evidence and practice review was undertaken over a period of 4 weeks. For the rapid evidence review, a comprehensive search of four databases (PubMed, CENTRAL (Cochrane), Web of Science and Google Scholar) was undertaken to identify published academic research regarding the effectiveness of approaches to optimise the use of emergency medical services. The search spanned the period 1 January 2012 to 5 September 2016. Two reviewers screened citations against pre-determined inclusion and exclusion criteria (Table 1) and data extraction of key information from included studies. The methodological quality of included systematic reviews was evaluated using the AMSTAR (Assessing the Methodological Quality of Systematic Reviews) tool, an 11-item tool with well-established validity and reliability that is extensively used to evaluate quantitative systematic reviews [13–15]. For narrative reviews, the key themes and conclusions drawn by the author were summarised.

**Table 1:** Rapid review inclusion and exclusion criteria

	Included	Excluded
Study type	Systematic reviews (quantitative and narrative) High quality primary studies	
Population	General public/public health campaigns Targeted over-users/mis-users Ambulance services or dispatch	Calls to Police or Fire or general emergency services
Study design	Observational or interventional	
Intervention	Public health campaigns Targeted interventions to problem subgroups Behavioural interventions Possibly dispatch or triage systems	
Outcomes	Reduced number of inappropriate or non-urgent calls to ambulance services or medical emergency phone numbers	
Publication status	English language Peer-reviewed journal publication or public reports Published in the last 5 years	

For the interviews, a semi-structured framework (Additional file 1) was used to enable interviewers to explore emerging themes as well as salient issues [16]. Participants were purposively selected based upon their experience or expertise in the use of Triple Zero emergency ambulance services or large-scale public health campaigns [17]. Interviews were audio-recorded and transcribed verbatim. Transcripts were then coded according to emergent themes and any relevant emerging topics [18] using NVivo10 (QSR International Pty Ltd. 2014).

#### Structured stakeholder dialogue

A structured stakeholder dialogue is a day-long, Chatham House Rules meeting of 15 to 20 individuals identified as being vested in the discussion topic. This includes operational-level and high-level (e.g. CEO, Manager, Professor) individuals representing consumers, government, professionals, researchers and other relevant stakeholders. Surfacing tacit knowledge from multiple perspectives enables the principle of collective problem solving to be harnessed, in which the knowledge of each individual contributes to a shared ‘team knowledge’ that can spark insights and generate action. This is by definition not possible to achieve by consulting each group independently.

### Results

#### Rapid evidence and practice review

The literature search yielded 2568 citations. Following screening, six reviews were eligible for inclusion, comprising five systematic reviews [19–23] and one narrative review [24]. Overall, review quality was high, indicating that a moderately high level of confidence can be placed in review findings. Five practice interviews were undertaken.

The evidence and practice review highlighted that definitions of ‘emergency’ and ‘appropriate use’ (critical to underpinning any campaign) were not definitive in research literature or practice. Different stakeholders, namely the medical fraternity, those experiencing medical difficulties and bystanders who observe them, bring unique, context-specific, behavioural and sometimes contrasting perspectives on what constitutes a medical emergency [24]. Reasons for inappropriate use of Triple Zero are therefore broad, reflecting this complexity. Although there are relatively few studies evaluating the effectiveness of strategies to reduce inappropriate emergency calls, there is some evidence to support provision of alternative services and secondary triage systems [21]. Financial incentives were found to be effective in the United States [21]; however, their transferability to Australia requires exploration. Mass media campaigns were identified as potentially effective but require careful planning [22].

Practice interviews reinforced and expanded upon the review findings. Ingredients of successful campaigns identified were a clear, evidence-informed strategy; raising the profile of an issue in the market; a strong, consistent and emotionally powerful campaign that is sustained over years; and co-ordination of the campaign with a strategically planned suite of behaviour change techniques such as legislation, enforcement and incentives. These factors enable multiple stakeholders to engage with the campaign from their particular context, including commissioning organisations, the media, government and leaders from the community and medical fraternity – another key requirement for success. Social norming is a powerful behaviour change device but can have unintended consequences if undesirable behaviours are inadvertently reinforced (e.g. see [25]). Campaigns need to be authentic and relevant, as lack of credibility is another key barrier to success.

Given these findings, careful consideration of the execution of a Triple Zero campaign was critical. A mass media campaign to the entire Victorian community offered the potential of considerable impact across the whole population and community support for legislative and other operational and policy measures designed to reduce inappropriate use of the service. However, such campaigns are not without risk. For example, messaging which emphasises the behaviour to be avoided can result in an increase in non-urgent calls because an undesirable behaviour is being socially normalised. Conversely, there is also a risk of a decrease in legitimate emergency calls. While a mass media campaign would need to be sustained over years to change behavioural norms, once changed, investment could be reduced. A campaign targeted at specific population cohorts could better focus on undesirable behaviours or typologies to immediately reduce inappropriate calls, but this option would have less impact on wider expectations and, therefore, give less support to other behaviour change measures. Targeted campaigns would also require continuous effort to maintain results. These considerations were fed into the subsequent stakeholder dialogue for deliberation.

#### Stakeholder dialogue

The dialogue was attended by 18 people who represented the Victorian Government, AV, hospitals, alternative ‘non-emergency’ health service providers, telecommunications authorities, media strategy, communications and behavioural research stakeholder groups.

Numerous and complex factors were identified during the dialogue regarding the issue of Triple Zero use for ambulances, including shifting of demand to other areas of the health service and addressing complex and interacting factors that drive calling behaviour. There was universal support for a media campaign as re-positioning the ambulance service and managing expectations of what it should and should not do.

The pros and cons of mass media and targeted campaign options were considered at length. It was acknowledged that a combination of mass media and targeted campaigns could be beneficial, but that a mass media campaign was a more desirable starting point. A mass media campaign could focus public attention on multiple health resources that, in combination, can address a broad spectrum of healthcare needs. It was agreed that awareness and attitude measures would be better initial reflections of campaign success (that is, within the first 12 to 18 months) than behaviour change (i.e. actual reduction in Triple Zero calls for non-emergencies), which would be a longer term measure. A range of success measures were identified in this context.

Consistent with the potential for unintended consequences described earlier, it was recognised that a critical consideration of any campaign is to not over-emphasise a focus on ‘what not to do’ (e.g. ‘don’t call Triple Zero if it’s not an emergency’) but frame positive messages around resources available for a broad range of healthcare needs, ranging from emergencies to information resources.

Full reports from the rapid evidence and practice review and stakeholder dialogue are available from the corresponding author on request.

## Phase 2 – Campaign and evaluation

### Methods

#### Campaign design

Based upon the formative research, a mass media campaign strategy was developed. It was agreed that a long-term campaign approach, involving two key stages as stated below, was required.

1. Sensitise – Help the community understand and accept why the issue presented is serious and legitimate; and
2. Educate – Once the community understands and agrees with the premise, provide the behavioural information the community needs to help solve the problem.

The aim of the first stage of the campaign was to foster an understanding that the primary role of ambulances is to attend to time-critical, life-threatening health emergencies. This approach is based on several models of behaviour change, such as the Theory of Planned Behaviour [26], which suggest that attitudes are a key predictor of behavioural intention, which in turn predicts actual behaviour. In other words, if individuals believe that ambulances should be used for emergencies only, they will be less likely to call Triple Zero for an ambulance in a non-emergency. To this end, the campaign sought to highlight the life-threatening consequences of ambulance resources being unavailable due to non-emergency calls to Triple Zero.

Five concepts were initially developed. Each concept was workshopped with members of the Victorian community in the state capital (Melbourne) and regional areas. All concepts received positive feedback across a range of evaluation criteria. However, one concept (subsequently titled *Will’s Story*) was most successful in achieving the required objectives. This concept was based upon the real-life story of a boy (Will) who required emergency ambulance services (including helicopter transport to the major Children’s Hospital in Victoria), told from the point of view of the paramedic. *Will’s Story* emphasised that it was not luck that saved his life but the fact the ambulance and paramedics were available because they were not otherwise occupied with non-emergencies.

Feedback from workshop participants in response to *Will’s Story* illustrated that:

- Even though the boy’s injury or illness was not mentioned, it was clear that his case was time critical and life threatening;
- The skill level and competence of the paramedics and emergency Mobile Intensive Care Ambulance and Air Ambulance services reinforced the excellence of the emergency services;
- People understood why such a high level of training and resources were required;
- The story generated a strong emotional connection and the twist on the reference to “*lucky to be alive*” was powerful and memorable; and
- It was evident that time was critical, and individuals should judge their situation before calling for an ambulance.

**Fig. 1:**
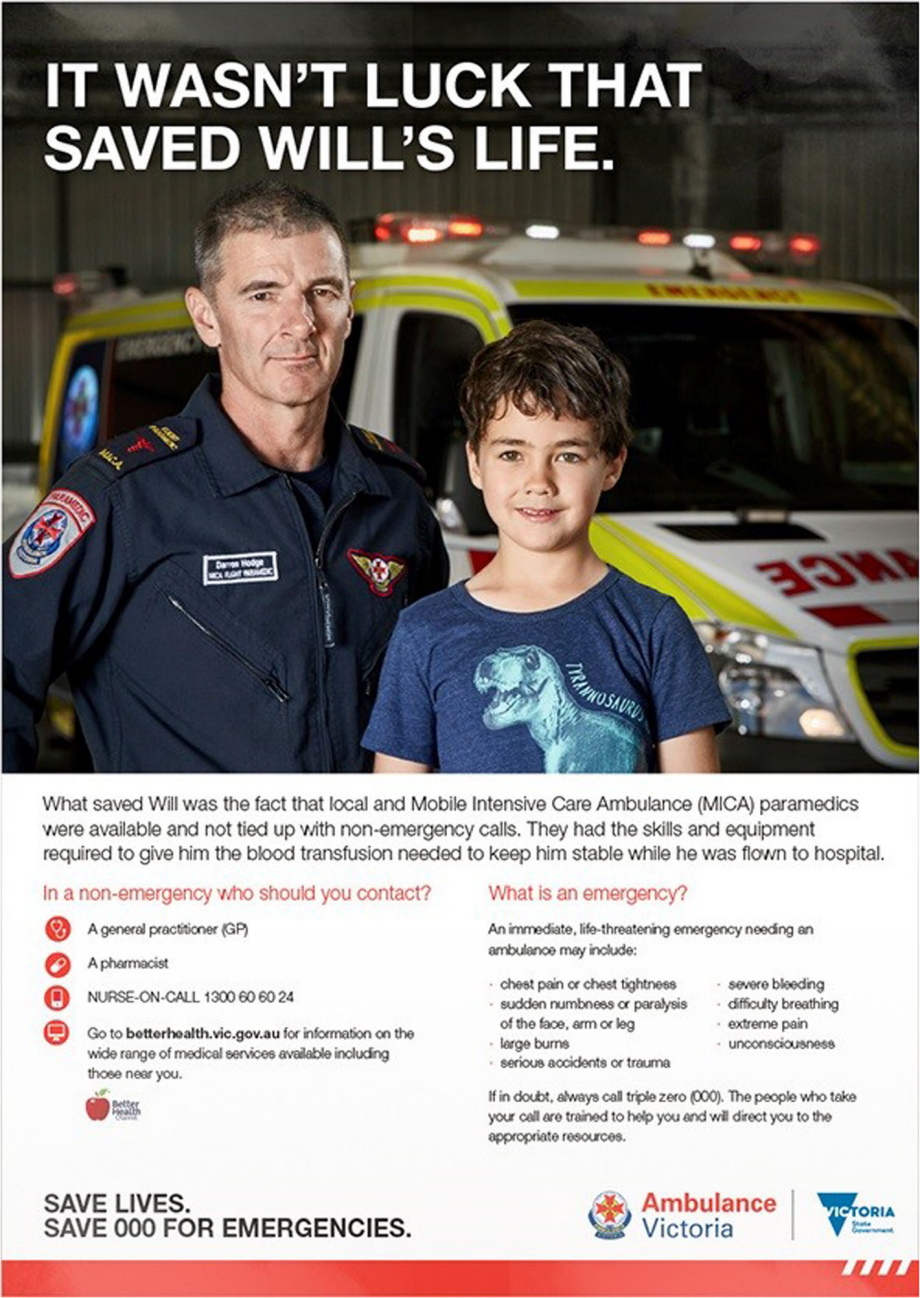
‘Save 000 for Emergencies’ campaign poster

The tag line for the campaign was “*Save Lives. Save 000 for Emergencies*.” *Will’s Story* ran from March until November 2017 (Fig. 1).

#### Monitoring and evaluation framework

It was recognised that any public campaign in this space must be subjected to robust monitoring and evaluation. The framework developed to monitor and evaluate the overall campaign programme encompassed four key metrics:

1. Campaign awareness,
2. Attitudes towards use of ambulance services,
3. Knowledge of alternative health services, and
4. Behaviour/use of emergency and non-emergency health services.

#### Survey data collection

To evaluate the effectiveness of the campaign in changing community-wide attitudes, a cross-sectional pre–post survey with ongoing tracking was conducted with three independent samples (one at each survey collection period). Survey data collection occurred in February 2017 (before the campaign commenced), June 2017 (4 months after the campaign commenced), and December 2017 (10 months after the campaign commenced).

#### Sampling

In order to achieve a representative sample of approximately 1000 respondents per survey round, an online panel research company was engaged to recruit participants and collect survey data. Eligible participants were members of the survey panel and included any member who lived in Victoria and was aged 18 years or older. Around 14,000 email invitations were distributed resulting in 1000+ completed surveys each round (approximately 8% response rate). The response rate was limited due to the use of quotas to facilitate stratified random sampling in order to obtain a sample that represented the Victorian adult population. Quotas were interlocking according to gender (male, female) and age (age bands 18–24, 25–34, 35–54, 55–69 and 70+), as well as geography (Melbourne, rest of Victoria). Panel members were randomly drawn from each strata (with a bias for younger male respondents who are known to have lower response rates) and quota groups were closed once the target number of completed surveys was achieved.

#### Measures

The data used in this study were collected as part of the overall campaign evaluation. From the larger evaluation, only relevant variables are included in this paper and are described below. These variables relate to metrics associated with (1) campaign awareness, (2) attitudes towards the use of ambulance services, and (3) knowledge of alternative health services.

Unprompted and prompted campaign awareness was measured by asking respondents if they could recall any advertisements about ambulance use, and if they recognised the TV advertisement for *Will’s Story*. Attitudes towards ambulance use were measured based upon the ‘Perceptions of Ambulance use’ scale [27]. The scale captures level of agreement with eight statements about ambulance use. While the original scale incorporated a five-point Likert scale, we adopted an 11-point scale (from 0 ‘Strongly disagree’ to 10 ‘Strongly agree’) to ensure consistency with similar agreement-type questions in the survey. Seven additional items, which had been used by the Victorian Government previously, were also added to the scale for comparative purposes. Knowledge of alternative health services was measured by asking respondents “Which of the following health services have you heard of before today?” The list included 20 Victorian health services, including Triple Zero, and an option for ‘Other’ and ‘None of the above’.

#### Analysis

The benchmark survey was administered in February 2017, before the campaign was launched, with 1037 participants (hereafter referred to as T1). The first evaluation survey was administered in June 2017, after the campaign had been active for 4 months, with 1052 participants (hereafter referred to as T2). The second evaluation survey was administered in December 2017, after the campaign had been active for 10 months, with 1018 participants (hereafter referred to as T3).

Data analysis was conducted using SPSS 23.0 (IBM). The modified ‘Perceptions of Ambulance use’ scale was assessed for factor structure and two latent factors were identified (see Additional file 1: Table S1 for factor loadings). Factor 1 included items related to the belief that ambulances should be for everyone to use regardless of their condition, it was labelled ‘Ambulances are for all’. Factor 2 included items related to the belief that ambulances are not an entitlement and should be reserved for emergency situations, it was labelled ‘Ambulances are for emergencies’. Analysis of Variance (ANOVA) and χ^2^ tests were also conducted to assess differences between the three survey rounds (T1, T2 and T3) and between respondents who were and were not aware of the campaign at T2 and T3.

### Results

Below is a summary of the evaluation findings. For a detailed explanation and outcomes of the evaluation data analysis see Additional file 1.

Unprompted awareness of the campaign remained relatively consistent between T2 (21.9%) and T3 (22.8%). Prompted awareness of the campaign increased significantly from 38.2% in T2 to 44.3% in T3 (*p* = 0.005). The mean score for the ‘Ambulances are for all’ subscale significantly decreased between T1 (M = 4.05) and T2 (M = 3.67, *p* < 0.001) and between T2 and T3 (M = 3.33, *p* = 0.003). The mean score for ‘Ambulances are for emergencies’ also increased significantly between T1 (M = 7.71) and T2 (M = 7.87, *p* = 0.042), however, it remained relatively unchanged in T3 (M = 7.88). On average, respondents recognised 7.25 Victorian health services in T1. The mean number of recognised services increased significantly in T2 (M = 7.64, *p* = 0.041) but remained relatively unchanged in T3 (M = 7.71). See Additional file 1: Table S2 for detailed statistical outcomes.

After aggregating respondents from T2 and T3 (who were exposed to the campaign material), there was a significant difference between those who were aware of the campaign and those who were not in terms of their attitudes towards ambulance use. ‘Ambulances are for all’ scores were significantly lower among those who recalled the campaign, either prompted (*p* = 0.001) or unprompted (*p* < 0.001), and ‘Ambulances are for emergencies’ scores were significantly higher among those who recalled the campaign, either prompted (*p* < 0.001) or unprompted (*p* < 0.001). See Additional file 1: Table S3 for detailed statistical outcomes. There was also a moderate negative correlation between ‘Ambulances are for all’ and service knowledge (*r* = −0.30, *n* = 3107, *p* < 0.001) and a small positive correlation between ‘Ambulances are for emergencies’ and service knowledge (*r* = 0.29, *n* = 3107, *p* < 0.001).

## Discussion

Public messaging around ambulance use needs to balance the need to reduce unnecessary calls against the risk of normalising the undesired behaviour or reducing genuine emergency calls. We attempted to achieve this balance in the current programme by drawing upon research evidence and by designing a thorough evaluation plan to track attitudes, awareness, knowledge and, ultimately, behaviour. This study offers an opportunity to examine how the application of the Forum approach can inform a state-wide campaign and population-level outcomes. The lessons drawn from the findings of this project will therefore be of value to policy-makers, emergency services and, more broadly, to the burgeoning field of behaviour change research.

The ‘Save Ambulances for Emergencies’ campaign is achieving its short-term attitudinal, awareness and knowledge goals. Attitudes towards ambulances being ‘for all’ decreased significantly between T1 (pre-campaign), T2 (4 months post introduction) and T3 (10 months post introduction), and attitudes towards ambulances being ‘for emergencies’ increased between T1 and T2. There was also a relationship between campaign awareness, attitude, and knowledge of other health services – indicating that those who were aware of the campaign and who were familiar with more health services were more likely to agree that ambulances should be used for emergencies only.

As proposed by the Theory of Planned Behaviour, in addition to attitudes, perceived behavioural control (the perceived ease or difficulty of performing a behaviour) is another antecedent of behavioural intent [26]. In the current study, those who lacked familiarity with appropriate non-emergency health services may harbour unfavourable attitudes towards ambulance use because of a lower level of perceived control over the appropriate behaviour – that is, it is easier for them to call Triple Zero because it is familiar. Given the relationship between attitude and knowledge of other services and the finding that knowledge did not change between T2 and T3, future campaigns could build on existing messaging while promoting alternate services for non-emergency conditions such as general practitioners, pharmacists, or health advice telephone services. Such an approach could address the issue of perceived behavioural control in addition to attitudes towards ambulance use – increasing the likelihood that behaviour change will occur.

While further research is required to determine if campaign activities will achieve the long-term goals of changing behaviour, these initial findings suggest that adopting a research-informed approach to campaign development has been successful in shifting attitudes towards ambulance use. Ongoing evaluations are highly recommended to ensure that the campaign messaging can be tested and improved over time.

### Limitations

As with any mass media behaviour change campaign, this project was subject to several limitations. First, a rapid review rather than a systematic review approach was taken in this project, as a systematic review was beyond the available scope and resources. A systematic review with more comprehensive review methods may have reached different conclusions. Second, the low response rate and use of an online panel (which typically adopt non-probability recruitment methods) can lead to sample bias [28]. To compensate for this, and to ensure that the samples broadly reflected the target population on key demographic characteristics, sampling quotas were applied each survey round. Third, while a pre–post study design was appropriate for evaluating the effectiveness of a mass media campaign, we cannot be certain that the changes observed in the survey were the direct result of the campaign (attribution). This is where ongoing monitoring and evaluation and a collaborative approach to intervention design and evaluation is crucial to help identify other activities that may have influenced the outcomes. While we are unaware of other external factors that may have influenced our results, we also cannot rule out some unknown influence. It is also worth recognising that attitude change does not always lead to behaviour change, although it is an important determinant of such change.

## Conclusions and future directions

Based on the findings from the formative and evaluation research phases, the campaign appears to have positively influenced community attitudes toward appropriate use of ambulances. Future iterations of the campaign should consider building upon this attitudinal message by supplementing it with behavioural control messaging around what services they should use in a non-emergency. Given the ultimate goal of the programme is to change behaviour, the next iteration of the evaluation should also shift from focusing on short-term outcomes of awareness and attitude towards long-term outcomes of actual behaviour, regarding the use of Triple Zero for an ambulance as well as the use of alternate health services.

## Additional file

Additional file 1: Interview framework for practice review. Campaign evaluation data analysis and results. (DOCX 25 kb)

## Abbreviation

AV: Ambulance Victoria

## Footnotes

1
http://www.premier.vic.gov.au/victorias-first-supercare-pharmacies-opening-soon
