# Supplementary material for: Ambulances are for emergencies: shifting attitudes through a research-informed behaviour change campaign
Source: Health Res Policy Syst. 2019 Mar 28;17:31. doi: 10.1186/s12961-019-0430-5 (PMC6437887; doi:10.1186/s12961-019-0430-5)
Supplement: Supplementary file 1 — Interview framework for practice review. Campaign evaluation data analysis and results. (DOCX 25 kb) [file 12961_2019_430_MOESM1_ESM.docx]

# Additional file 1

## Appendix 1: Interview framework for practice review

The interviews were semi-structured, allowing the interviewers to explore emerging themes as well as salient issues (Spencer et al., 2003) The interview framework was as follows:

1. Can you provide a brief introduction and outline your role in the Triple Zero emergency ambulance service in Victoria, including how long you have been in this role?
2. From your perspective and experience, what are key issues that need to be addressed to optimise the Triple Zero emergency ambulance service in Victoria?
3. What strategies are you aware of that have been employed in the past to optimise the Triple Zero emergency ambulance service in Victoria?
   1. (if answered 3) How successful have these strategies been?
   2. (if answered 3a) What factors do you think have contributed to the success or failure of previous strategies?
4. If you could change one thing about the Triple Zero emergency ambulance service in Victoria - not just to do with your role but anything at all - what would it be?
5. Do you have any other comments on the Triple Zero emergency ambulance service in Victoria?

*For participants with no working knowledge of the Triple Zero system, but have been involved in a successful public campaign:*

1. Can you provide a brief introduction and outline your role in [name of campaign] in Victoria?
2. What factors do you think contributed to the success of [name of campaign]?
3. What barriers to success did you encounter, and how did you address these?
4. What would you do differently?
5. Based on these reflections, how would you approach development of a campaign to optimise appropriate use of the Triple Zero emergency ambulance service in Victoria?
   1. (if not addressed in 5) Who would you involve? How would you bring all stakeholders with you? What wider contextual factors would you consider?
6. Do you have any other comments?

## Appendix 2: Campaign evaluation data analysis and results

### Data analysis

A principal component analysis with Varimax rotation was conducted on the Perceptions of Ambulance use scale. Two latent factors were identified with Eigenvalues greater than 1. These factors accounted for 48.6% of the variance in attitudes. Item 7, “Patients get a higher priority in the hospital if they arrive in an ambulance” did not load above .40 on either factor and was removed from subsequent analyses. All other items loaded above .40 with minimal cross-loadings. Mean scores were calculated for each factor across the relevant items and were used in subsequent analyses.

As found by Toloo et al. (2013), Factor 1 included items 1-4 from the original scale, as well as items 9, 11, 13 and 14 from the additional items. All items in Factor 1 loaded above .70 (see Table S1 for individual item factor loadings) and were related to the belief that ambulances should be for everyone to use regardless of their condition. Factor 1 was labelled ‘Ambulances are for all’ (α=.90). Factor 2 included items 5, 6, 8, 10, 12, and 15 with factor loadings between 0.485 and .656. After conducting a reliability analysis, item 10 (“It is acceptable to wait for an ambulance if you are not critically sick”) was also removed from subsequent analysis due to poor inter-item correlation. The remaining items in Factor 2 related to the belief that ambulances are not an entitlement and should be reserved for emergency situations, it was labelled ‘Ambulances are for emergencies’ (α=.65 – after removing item 10).

Table S1. Perceptions of Ambulance use scale: exploratory factor analysis (factor loadings) (15-items).

| **#** | **Item** | **Ambulances are for all** | **Ambulances are for emergencies** |
| --- | --- | --- | --- |
| 3 | People should be able to use the ambulance if they can’t afford a taxi no matter how critical their condition is | .782 |  |
| 4 | People should use the ambulance if they can’t access other means of transport regardless of the seriousness of their condition | .781 |  |
| 9 | People who pay for Ambulance membership deserve an ambulance to be sent if they call for one regardless of how serious their condition is | .779 |  |
| 1 | Ambulances are for everyone to use when they feel unwell | .762 |  |
| 13 | Ambulances can be used when the doctor isn’t available | .754 |  |
| 14 | It is OK to call for an Ambulance if you are embarrassed and don’t want to seek treatment outside of the home | .751 |  |
| 2 | Everyone is entitled to ambulance services regardless of how serious their condition is | .747 |  |
| 11 | Our taxes pay for ambulances so we deserve to be sent one if we call for one | .723 |  |
| 6 | Using an ambulance for a non-emergency condition is a misuse of the system |  | .656 |
| 12 | Ambulances are not a free ride into hospital |  | .640 |
| 5 | People should call the ambulance only if it’s an emergency |  | .638 |
| 15 | It is OK for someone to be referred to another service if they call for an ambulance but it isn’t an emergency |  | .605 |
| 8 | We expect too much from paramedics and the ambulance system |  | .511 |
| 10~ | It is acceptable to wait for an ambulance if you are not critically sick |  | .485 |
|  | Total % of variance explained | 48.6% | |

~This item was removed from subsequent analyses.

In addition, Analysis of Variance (ANOVA) were conducted to determine differences in mean attitude scores, and in the mean number of recognised services between the three survey rounds (T1, T2, and T3). Chi-square tests were also used to test differences in the proportion of respondents between survey periods who were aware of the campaign (prompted and unprompted) and the proportion who could describe the correct campaign message as ‘save 000/ambulances for emergencies’.

### Results

*Changes between survey rounds*

Unprompted awareness of the campaign remained relatively consistent between Waves 1 (21.9%) and 2 (22.8%) [χ^2^ (1, n=2,070) = 0.26, *p*=.613]. Prompted awareness of the campaign video advertisement increased significantly from 38.2% in T2 to 44.3% in T3 [χ^2^ (1, n=2,070) = 7.92, *p*=.005]. In addition, after viewing the campaign 62.5% of respondents in T3 correctly described the key message as ‘Triple Zero/Ambulances are for emergencies’.

Table S2 shows the attitudes towards use of ambulance services and results of the ANOVA tests. While the mean score for the ‘Ambulances are for All’ subscale was already relatively low during T1 (M=4.05, SD=2.26), this score significantly decreased between T1 and T2 (M=3.67, SD=2.28; *p*<.001) and between T2 and T3 (M=3.33, SD=2.41; *p*=.003). In contrast, the mean score for ‘Ambulances are for Emergencies’ was relatively high during T1 (M=7.71, SD=1.52). This score also increased significantly between T1 and T2 (M=7.87, SD=1.49; p=.042), however it remained relatively unchanged in T3 (M=7.88, SD=1.61; p=.982).

Also presented in Table S2 is an overview of the mean number of health services recognised by respondents in each survey round. On average, respondents recognised 7.25 (SD=3.62) Victorian health services in T1 – Ambulance Victoria (84.7%) and Triple Zero (000) for an ambulance or paramedic (81.4%) were the most well-recognised services. The mean number of recognised services increased significantly in T2 (M=7.64, SD=3.62; p=.041) but remained relatively unchanged in T3 (M=7.71, SD=3.73; p=.889).

Table S2. Perceptions of Ambulance use and knowledge of health services descriptive statistics

|  | Mean | SD | 95% CI | | N | F | *p* value |
| --- | --- | --- | --- | --- | --- | --- | --- |
| *'Ambulances are for All'* |  |  |  | |  |  |  |
| T1 | 4.05 | 2.26 | 3.91- | 4.19 | 1037 | 24.83 | 0.000 |
| T2 | 3.67 | 2.28 | 3.53- | 3.81 | 1052 |  |  |
| T3 | 3.33 | 2.41 | 3.18- | 3.48 | 1018 |  |  |
| *'Ambulances are for Emergencies'* |  |  |  |  |  |  |  |
| T1 | 7.71 | 1.52 | 7.61- | 7.80 | 1037 | 4.14 | 0.016 |
| T2 | 7.87 | 1.49 | 7.78- | 7.96 | 1052 |  |  |
| T3 | 7.88 | 1.61 | 7.78- | 7.98 | 1018 |  |  |
| *Number of recognised Victorian health services* |  |  |  |  |  |  |  |
| T1 | 7.25 | 3.62 | 7.03- | 7.47 | 1037 | 4.77 | 0.009 |
| T2 | 7.64 | 3.75 | 7.41- | 7.87 | 1052 |  |  |
| T3 | 7.71 | 3.73 | 7.48- | 7.94 | 1018 |  |  |

*Relationship between awareness, attitude and knowledge*

At a total sample level (aggregating respondents in T2 and T3) there was a significant relationship between awareness of the campaign and attitudes towards ambulance use. As shown illustrated in Table S3, ‘Ambulances are for All’ scores were significantly *lower* among those who recalled the campaign either prompted (p=.001) or unprompted (p<.001). ‘Ambulances are for Emergencies’ scores were significantly *higher* among those who recalled the campaign either prompted (p<.001) or unprompted (p<.001). Similarly, the relationship between attitude towards ambulance use and knowledge of health services was measured using Pearson product-moment correlation coefficient. There was a moderate negative correlation between ‘Ambulances are for All’ and service knowledge (*r* = -.30, n=3,107, p<.001). There was also a small positive correlation between ‘Ambulances are for Emergencies’ and service knowledge (*r* = -.29, n=3,107, p<.001).

Table S3. Attitude scores by unprompted and prompted awareness (No/not sure, Yes) of advertising about ambulance use.

|  | Mean | SD | 95% CI | | N | F | *p* value |
| --- | --- | --- | --- | --- | --- | --- | --- |
| *'Ambulances are for All'* |  |  |  |  |  |  |  |
| Unprompted awareness - No/not sure | 3.59 | 2.30 | 3.48- | 3.71 | 1608 | 10.76 | 0.001 |
| Unprompted awareness - Yes | 3.19 | 2.51 | 2.96- | 3.42 | 462 |  |  |
| Prompted awareness - No/not sure | 3.75 | 2.34 | 3.62- | 3.88 | 1217 | 33.69 | 0.000 |
| Prompted awareness - Yes | 3.15 | 2.33 | 2.99- | 3.30 | 853 |  |  |
| *'Ambulances are for Emergencies'* |  |  |  |  |  |  |  |
| Unprompted awareness - No/not sure | 7.79 | 1.55 | 7.71- | 7.87 | 1608 | 21.73 | 0.000 |
| Unprompted awareness - Yes | 8.17 | 1.51 | 8.03- | 8.31 | 462 |  |  |
| Prompted awareness - No/not sure | 7.68 | 1.59 | 7.60- | 7.77 | 1217 | 45.37 | 0.000 |
| Prompted awareness - Yes | 8.15 | 1.45 | 8.05- | 8.24 | 853 |  |  |
